# Autonomic Dysregulation in Multiple Sclerosis

**DOI:** 10.3390/ijms160816920

**Published:** 2015-07-24

**Authors:** Alexandra Pintér, Domonkos Cseh, Adrienn Sárközi, Ben M. Illigens, Timo Siepmann

**Affiliations:** 1 Institute of Human Physiology and Clinical Experimental Research, Faculty of Medicine, Semmelweis University, Budapest 1085, Hungary; E-Mails: Cseh.Domonkos@med.semmelweis-univ.hu (D.C.); Sarkozi.Adrienn@med.semmelweis-univ.hu (A.S.); 2 Center for Clinical Research and Management Education, Division of Health Care Sciences, Dresden International University, Dresden 01067, Germany; 3 Department of Neurology, Beth Israel Deaconess Medical Center, Harvard Medical School, Boston, MA 02215, USA; E-Mail: illigens@bidmc.harvard.edu; 4 Department of Neurology, University Hospital Carl Gustav Carus, Technische Universität Dresden, Dresden 01307, Germany; 5 Department of Psychotherapy and Psychosomatic Medicine, University Hospital Carl Gustav Carus, Technische Universität Dresden, Dresden 01307, Germany

**Keywords:** multiple sclerosis, autonomic, orthostatic dysregulation, bladder, gastrointestinal, dysfunction

## Abstract

Multiple sclerosis (MS) is a chronic, progressive central neurological disease characterized by inflammation and demyelination. In patients with MS, dysregulation of the autonomic nervous system may present with various clinical symptoms including sweating abnormalities, urinary dysfunction, orthostatic dysregulation, gastrointestinal symptoms, and sexual dysfunction. These autonomic disturbances reduce the quality of life of affected patients and constitute a clinical challenge to the physician due to variability of clinical presentation and inconsistent data on diagnosis and treatment. Early diagnosis and initiation of individualized interdisciplinary and multimodal strategies is beneficial in the management of autonomic dysfunction in MS. This review summarizes the current literature on the most prevalent aspects of autonomic dysfunction in MS and provides reference to underlying pathophysiological mechanisms as well as means of diagnosis and treatment.

## 1. Introduction

In developed countries, multiple sclerosis (MS) is the most prevalent chronic neurological disorder in young individuals, affecting over 400,000 persons in the United States alone [1]. Autonomic nervous system disturbances including sweating abnormalities, urinary dysfunction, orthostatic dysregulation, gastrointestinal symptoms and sexual dysfunction are frequent complications that reduce the quality of life of affected patients [2,3]. Urogenital symptoms occur in the vast majority of all MS sufferers at some point during the course of the disease. Multimodal and interdisciplinary strategies of diagnosis and treatment of autonomic dysfunction can improve health and quality of life. While effective pharmacological and non-pharmacological therapies are available to alleviate several autonomic symptoms in MS, the underlying pathomechanisms have not yet been fully elucidated and causative treatment is still lacking [3,4,5,6]. Moreover, the way autonomic function should be quantitatively assessed in MS patients in clinical practice has not yet achieved a consensus which would allow for a standardized recommendation and help evaluate response to neuroprotective and disease-modifying therapies. This review summarizes the current literature on autonomic dysfunction in MS with a focus on pathophysiological mechanisms, diagnostic techniques and treatment strategies.

## 2. Search Methods and Study Selection Criteria

We performed a narrative review which intended to provide a summary of the literature and guide to the clinician. We searched MEDLINE using the PubMed interface. The text words “autonomic” and “multiple sclerosis” with the use of the Boolean operator “AND” were used to identify relevant studies that examined the association between autonomic disturbances and multiple sclerosis. In a first literature research we exclusively chose these two text words as well as the abbreviation “MS” and their corresponding Medical Subject Heading (MeSH) terms (“autonomic” AND “multiple sclerosis” OR “autonomic” AND “MS”) to ensure maximum coverage of all potentially eligible articles stored in the searched electronic database. Additionally, we performed a second literature search using the same electronic database using more specific MeSH terms to ensure coverage of all specific aspects our review focused on. For this purpose we established a search strategy using the following MeSH terms and their combinations: “multiple sclerosis” OR “MS” AND “autonomic”, AND “dysregulation”; “multiple sclerosis” OR “MS” AND “autonomic” AND “dysfunction”; “neurogenic lower urinary tract dysfunction” OR “NLUTD”; “neurogenic lower urinary tract dysfunction” OR “NLUTD” AND “multiple sclerosis” OR “MS”; “multiple sclerosis” OR “MS” AND “detrusor overactivity” OR “detrusor sphincter dyssynergia”; “multiple sclerosis” OR “MS” AND “cardiovascular” OR “orthostatic” OR “syncope” OR “POTS” OR “postural orthostatic tachycardia syndrome”; “multiple sclerosis” OR “MS” AND “gastrointestinal” OR “anorectal” OR “bowel” OR “dysphagia”, OR “sexual” OR “erectile dysfunction” OR “genital”; “multiple sclerosis” OR “MS” AND “sudomotor”; “sudomotor” AND “axon reflex”; “multiple sclerosis” OR “MS” AND “pupillomotor”. Additionally we combined those of the forementioned MeSh terms that refer to symptoms of autonomic dysfunction in MS with the MeSh terms “assessment” OR “diagnosis” OR “treatment” OR “therapy” using the Boolean operator “AND” to achieve coverage of papers relevant to diagnosis and treatment. We included epidemiological data, prospective controlled clinical trials, and retrospective database analyses conducted from 1953 to 2015. In addition, we manually searched the bibliographies of recently published opinion statements, reviews, and meta-analyses.

## 3. Neurogenic Lower Urinary Tract Dysfunction

### 3.1. Epidemiology

Neurogenic lower urinary tract dysfunction (NLUTD) is a frequent complication of MS which reduces quality of life [7,8] and affects 360,000 MS patients in the United States alone [1,9]. Bladder dysfunction is among the most frequent neurological manifestations of MS. Other relevant neurological symptoms include motor dysfunction, cerebellar, brainstem, sensory, visual and mental impairment. The estimated prevalence of urinary symptoms varies depending on the duration and severity of neurological deficiency and disability [9]. A recent study found that 92% of 1047 MS patients reported at least one lower urinary tract symptom. The most common symptoms were post micturition dribble (64%), urinary urgency (62%), feeling of incomplete emptying (61%) [9]. An ancillary analysis of the North American Research Committee on Multiple Sclerosis (*n* = 9702) reported that 65% of MS patients suffered from at least one moderate-to-severe urinary symptom (frequency, urgency, nocturia, leakage) [10]. In a meta-analysis of 22 published studies of symptomatic MS patients (total *n* = 1882), detrusor overactivity (DO) was detected in 62%; hypocontractility in 20% and detrusor sphincter dyssynergia (DSD) occurred in 25% of the patients [11]. However, most MS patients have a combination of these urological conditions.

### 3.2. Clinical Features and Complications

NLUTD patients may exhibit storage (e.g., urgency, daytime frequency, nocturia, urge urinary incontinence) or voiding symptoms (e.g., slow stream, intermittent stream, hesitancy, incomplete emptying) or combinations of these (including paradoxal urgency/hesitancy, intermittent urgency followed by subsequent inability to start voiding) [12].

In patients with NLUTD, urodynamic evaluation reveals functional abnormalities in the background of these clinical symptoms. The etiology of urinary storage problems in MS was shown to be DO, while irregular emptying is due to detrusor underactivity. DSD is frequently present in patients with combined voiding and storage symptoms. The underlying mechanism of DSD is constituted by involuntary detrusor contractions against the closed internal and/or external sphincter resulting in elevated post voiding residual (PVR) volumes and vesicourethral reflux [13,14]. If left untreated, NLUTD may cause further complications and irreversible changes in the upper and lower urinary tract, such as bladder calculi, hydronephrosis, urinary tract infections (UTI) and chronic renal failure [11,15].

Bladder dysfunction is usually reported in association with another neurological dysfunction, particularly pyramidal motor symptoms and usually occurs in the early stages of MS [16]. The severity of lower urinary tract symptoms in MS patients is related to the level of walking disability [5,17]. Therefore, motor symptoms of the extremities in MS patients should raise the physician’s awareness of possible bladder dysfunction, and *vice versa*.

### 3.3. Radiological Findings

Due to the demyelinizing nature of the disease, structural myelin damage of the neuronal connections between centers regulating lower urinary tract function compromise functional integrity of the neurourinary system in MS. There is a clinicoradiological paradox in MS patients: only low-moderate association is shown between clinical disabilities and the burden represented by the amount of lesional tissue on the magnetic resonance imaging (MRI) [18]. The morphological abnormalities, underlying NLUTD are diverse. Detrusor overactivity is often associated with suprasacral lesions found in the medial frontal lobe cortex, cerebellum, insula, dorsal midbrain, periaqueductal gray, pons micturition center [19,20]. Medullary lesions between the pontine and sacral micturition centers, especially cervical spinal lesions may cause urethral dysfunction, *i.e.*, disturbances of urine storage and bladder emptying, with DSD is the clinically most pronounced defect [11,20]. Sacral plaques and peripheral nerve demyelinization are less common than suprasacral lesions. Sacral and peripheral lesions have been suggested to inhibit facilitated detrusor contraction leading to acontractile or weak detrusor contraction and urinary retention [21].

### 3.4. Diagnosis

Early diagnosis and initiation of treatment in MS related NLUTD are pivotal to increase quality of life and slow progression of the symptoms [22]. International management guidelines, developed by the International Continence Society, the American Urological Association and the European Association of Urology exist for NLUTD in general, but not for MS patients specifically [23,24,25]. However, several countries, *i.e.*, France, UK, Italy and Turkey, have also developed national MS specific management guidelines for NLUTD [5,15,26,27,28]. Considerable differences exist between these guidelines, particularly regarding diagnosis and follow-up. Treatment recommendations also vary among these guidelines, especially with respect to the requested degree of specialization of the treating physician. However, a consensus appears to be present on the recommendation that initial treatment of MS patients with NLUTD can be provided by a general practitioner/neurologist and a reasonable non-invasive evaluation including a detailed history, physical examination, complete urine analysis, serum creatinine level, voiding diary, urinary system ultrasonography, urine flow rate and importantly PVR urine measurement [28,29]. In cases of initial conservative treatment failure and/or upper urinary tract deterioration, invasive urodynamic tests should be applied. Red flags were introduced to refer directly the patient to a specialized neuro-urology team such as more than 3 symptomatic urinary tract infections or severe urinary tract infection with fever in the previous year, lumbar pain during voiding, immunotherapy, Expanded Disability Status Scale (EDSS) superior to 6, male older than 55 years, and significant ultrasound abnormalities or PVR superior of 100 mL [30,31].

### 3.5. Conservative Therapy

In MS patients, the management of NLUTD may be complicated by the progressiveness of the disease and the presence of other disease-specific manifestations, which can adversely influence treatment outcomes. It is recommended to tailor the management of NLUTD in these patients to the patient’s current needs, mobility, compliance and the level of disability [5].

For patients with mild disability from MS, physical interventions such as pelvic floor muscle training and behavioral treatment may be beneficial. However, the cognitive status of the patient should be taken into account when determining the optimal behavioral management program [32].

For MS patients with raised PVR (over 100–150 mL), local and international guidelines describe clean intermittent (self) catheterization (IC, ISC, respectively) as the mandatory symptomatic treatment of choice [5,24,25,27]. The use of indwelling catheters is only considered to be an option if IC is no longer possible because of the risk of UTIs and other complications [5,25]. If there is no significant PVR, antimuscarinic oral drug is the symptomatic treatment of choice. However, evidence is lacking for the efficacy of anticholinergic drugs in the treatment for DO in MS patients. A systematic review investigated the effects of anticholinergic treatment in MS patients [33]. Only those randomized, cross-over trials were included in this review that were either placebo-controlled or compared two or more medical treatments. The authors concluded that anticholinergic drugs are inefficient to treat urinary dysfunction in MS patients. Furthermore, usage of antimuscarinics has been associated with increased risk of urinary retention, UTIs, and constipation [5,27]. Their central nervous system related side effects include deterioration of already impaired cognition and memory in MS patients. Antimuscarinics that do not cross the blood brain barrier and those antimuscarinics with selective affinity for the M3 receptor are recommended [34,35].

It has been suggested that a combination of IC and antimuscarinic treatment could be used in MS patients to control both bladder overactivity and emptying problems [5,27]. Alpha-blocking agents were shown to improve emptying problems without significant PVR [36,37]. However, randomized controlled clinical studies are needed to elucidate the safety and efficacy of α-blockers in patients with MS and lower urinary tract symptoms. It is advised to initiate a new evaluation at 3 months post treatment onset to assess efficacy of treatment and PVR. In case of insufficient efficacy or elevated PVR, the patient must be referred to specialists [26].

As second line medical treatments for MS patients guidelines mention the option of using desmopressin for the treatment of DO problems such as daytime frequency or nocturia [28]. Despite some evidence from prospective trials of its short-term efficacy, there are no studies on the long-term use of desmopressin to date. When using desmopressin, the risk of hyponatremia and fluid retention should be considered [38].

Cannabinoids, e.g., nabiximol might provide some benefits in the treatment of DO symptoms of patients with MS, they are regarded as an adjunctive treatment, when antimuscarinics and other treatment modalities were inadequate [39,40]. However, further randomized controlled trials providing higher evidence levels are needed to evaluate the safety and efficacy of cannabinoids in MS bladder dysfunction treatment.

Therapy with low doses of antibiotics is suggested to diminish the risk of developing UTIs [27]. Randomized, double-blind, placebo-controlled study has also shown the efficacy of cranberry extract tablets in the prevention of UTIs [41].

### 3.6. Invasive Treatment

A minimally invasive therapy that can be used to treat neurogenic DO is intravesical injection with botulinum-A neurotoxin (BoNT-A) [42,43]. Refractory DO in patients with MS was one of the primary indications of this intervention [44]. The main effect of BoNT-A is the relaxation of the detrusor musculature by antagonizing the acetylcholine receptors. Studies with intravesical BoNT-A injections have shown both clinical and urodynamic amelioration and an improving quality of life. These beneficial effects were to be observed for about 8–10 months on average post-BoNT-A [42,45]. OnabotulinumtoxinA, a BoNT-A product class has been approved by the US Food and Drug Administration for the treatment of NLUTD in patients with MS (or spinal cord injury) largely based on two phase 3 randomized, placebo-controlled studies in patients with NLUTD due to MS (*n* = 381) or spinal cord injury (*n* = 310) [46,47]. OnabotulinumtoxinA was generally well tolerated; UTI (24%) and urinary retention (17%) were its noteworthy side effects. Therefore, PVR monitoring and at critical retention volume, ISC is recommended [48]. Intravesical BoNT-A injections may also be an option for end-stage MS patients and for patients with indwelling suprapubic and urethral catheters who develop chronic urethral leakage of urine [5,49]. Few studies noted, that BoNT-A injections into the external urethral sphincter decreased detrusor and urethral pressures, reduced PVR in patients with DSD, but this could not be confirmed in a randomized controlled trial in MS patients [50,51].

Chemical neuromodulation by vallinoids, capsaicin and resiniferatoxin are another treatment option for NLUTDs [52,53].

Peripheral tibial nerve stimulation represents another minimally invasive approach. A prospective non placebo-controlled trial of 83 MS patients with DO refractory to medical therapy demonstrated that 89% had at least 50% improvement in symptoms after peripheral tibial nerve stimulation that lasted for 2 years on average. There were significant decreases in daytime frequency, nocturia, and improvements on urodynamic parameters. Further studies are warranted to confirm these results in larger populations of patients [54].

Sacral neuromodulation is another minimally invasive treatment which has been shown to be effective in patients with non-neurogenic DO. If neurogenic DO symptoms are refractory to less invasive treatments this therapy may be considered [5]. Minardi *et al.* have found that sacral neuromodulation is effective in the treatment of voiding dysfunction in patients with MS in a medium to long-term follow-up [55]. The treatment should be indicated to MS patients with refractory urgency urinary incontinence or MS with urinary retention due to DSD. Sacral neuromodulation is provided by experienced centers.

Patients with MS, urinary incontinence, and urinary upper tract deterioration refractory to conservative and minimal invasive treatment options are candidates for surgical treatment. This should be undertaken in experienced centers to minimize anesthesiological and surgical risks [5]. Operational treatment alternatives such as bladder augmentation, enterocystoplasty and urinary diversion gave optimal results in stress, complex or refractory urinary incontinence, catheter intolerance and/or neurogenic DO due to MS [5,24,25]. Also patients with severe stress urinary incontinence due to catheter-induced trauma to the urethral sphincter may benefit from the surgical intervention (e.g., formal urethral closure with bladder drainage via a suprapubic catheter, ileal conduit with simultaneous removal of the bladder or formation of a vesico-vaginal fistula).

## 4. Cardiovascular Dysregulation

### 4.1. Epidemiology

Cardiovascular autonomic dysfunction is present in large proportion of patients with MS [56,57]. Prevalence data vary among studies, in parts due to heterogeneity in the way cardiovascular autonomic dysfunction was defined in these studies [56,57]. In a recent meta-analysis, Racosta *et al.* synthesized data of 16 studies with 611 MS patients either with relapsing-remitting or progressive forms examined by at least three cardiovascular autonomic tests. According to their results, cardiovascular autonomic dysfunction was present in 42% of included patients based on the definition of minimum one pathological test result, whereas prevalence was as low as 19% when using the definition of at least two abnormal cardiac autonomic tests [58].

### 4.2. Clinical Features

The main clinical symptoms connected with cardiovascular autonomic neuropathy are fatigue [59,60] and pathological responses to orthostatic challenge such as syncope, palpitation, dizziness, nausea, general weakness, hot flashes and sweating [61,62,63,64,65,66]. Symptoms associated with orthostatic dysregulation occur in up to 63% of the patients [61,62,63,64,65,66]. Postural orthostatic tachycardia syndrome (POTS), vasovagal syncope and orthostatic hypotension can be induced by tilt table test in MS patients [64]. Interestingly, the prevalence of syncope is higher in patients in remission, while POTS appears more frequently in patients in relapse [64]. Newly onset cardiac arrhythmias [67,68,69] can be the consequences of deteriorated cardiac autonomic regulation caused by MS.

### 4.3. Pathomechanisms

Both sympathetic and parasympathetic parts of the cardiovascular autonomic system are affected by MS [57,70,71,72,73,74,75,76,77]. An association was found between sympathetic dysfunction and clinical activity of MS [78]. Parasympathetic abnormalities seem to be linked to progression of disability as measured by the EDSS [78]. Consistently, longer duration of MS has been linked to progressive deterioration of parasympathetic regulation [77]. In line with these findings, serum norepinephrine and epinephrine levels were shown to be lower in clinically active relapsing-remitting patients than in clinically stable patients [78]. Keller *et al.* also found decreased plasma norepinephrine levels in relapsing-remitting multiple sclerosis patients [71]. Supine serum norepinephrine level was found to be increased in chronic progressive patients indicating sympathetic dysregulation [79]. According to Flachenecker *et al.* impairment in parasympathetic regulation could be the consequence of MS, however damaged sympathetic function may have a pathogenetic role in the development of MS [78]. Total midbrain lesion volume and total parietal lesion volume (to a lesser degree) showed association with cardiovascular autonomic abnormalities [72]. In line with these findings, other groups identified MS lesions in the brainstem as the feasible morphological substrates of cardiovascular autonomic dysfunction [56,61]. According to the findings of de Seze *et al.*, spinal axonal loss is a stronger determinant of autonomic disturbances than demyelination. They found pronounced association between the reduction of spinal cord cross sectional area and autonomic dysfunction, while they observed no relation between the number and location of spinal MS lesions and autonomic abnormalities [80].

### 4.4. Diagnosis

In MS, heart rate responses to the Valsalva maneuver, deep breathing, orthostatic challenge (tilt table test or active change of posture) are the most widely used tests in clinical practice for the assessment of predominantly parasympathetic function, while predominantly sympathetic function can be estimated by the measurement of blood pressure alterations caused by change in posture and sustained handgrip [78,80,81]. More subtle deterioration of cardiovascular autonomic function can be assessed in MS patients by more sophisticated laboratory tests including measurement of heart rate variability in the time or frequency domain [59,77,82,83], estimation of baroreflex function [75,84], examination of muscle sympathetic nerve activity [71]. Cutaneous axon reflex assessment appears to be a promising autonomic testing modality in central neuronal diseases such as MS [85,86].

### 4.5. Therapy

Since no “one size fits all” solution is available to treat cardiovascular autonomic disturbances, multimodal and individualized treatment strategies are desirable. After the assessment of the nature of sympathovagal imbalance with the aforementioned tests, there are non-pharmacological, pharmacological and surgical strategies to treat cardiovascular dysfunction in MS patients.

Reduced fat intake within the confines of Mediterranean diet can enhance parasympathetic function [87,88]. Adequate hydration can prevent fainting episodes [4] and appropriate water intake can augment sympathetic activity [89]. Mild to moderate intensity aerobic exercise may have beneficial effects in patients with decreased vagal activity [88,90]. Short bursts of high intensity aerobic exercise may enhance sympathetic functions in patients with lower-than-normal sympathetic activity [88,91]. Head-up tilt sleeping position can improve sympathetic activity in patients with sympathetic disturbances [92]. Patients with orthostatic intolerance may benefit from therapy with volume expanders, vasoconstrictor agents and acetylcholinesterase inhibitors [93,94,95]. Sympathomimetic drugs like β-adrenergic agonists, α1-adrenergic agonists and α2-adrenergic antagonists may play an important role in the treatment of patients with lower sympathetic activity [88,96,97]. Interestingly, intermittent use of low-dose nicotine as moist snuff or dry snuff may have favorable effects by upregulating sympathetic functions [88]. Recently, droxidopa, an oral norepinephrine precursor, was shown to improve symptomatic neurogenic orthostatic hypotension [98]. In fact, this open-label dose optimization trial, followed, in responders, by 7-day washout and a consecutive 7-day double-blind study of droxidopa *vs.* placebo provided Class I evidence that droxidopa improves neurogenic orthostatic hypotension in responders. As the result of recombinant erythropoietin treatment, serum norepinephrine level may increase and the responsiveness of the vessel wall to norepinephrine may improve [88,99]. A possible link between chronic cerebrospinal venous insufficiency and MS has been previously discussed but a recent observational case-control study provided strong evidence against this conception [100,101,102,103,104].

## 5. Gastrointestinal Dysregulation

### 5.1. Epidemiology

The area of bowel dysfunction is considered the “Cinderella” of MS research, although 40%–81% of the patients complain about functional loss in the gastrointestinal domain [66,105]. The most frequently reported gastrointestinal (GI) symptoms encompass anorectal dysfunction characterized by constipation (37%–68%) [105,106], fecal incontinence (15%–51%) [105,107] or the combination of these (23%) [108] and deglutitive problems such as dysphagia (21%) [105]. Similarly to bladder and sexual dysfunctions, GI disturbances seem to affect quality of life with a considerable negative impact [109,110]. Bowel dysfunction can be a source of severe psychosocial disabilities, by limiting the capability of work or engaging in social interactions [111].

### 5.2. Clinical Symptoms and Associations

Levinthal *et al.* investigated GI symptoms in a sample of 218 MS patients [105]. Besides common constipation, fecal incontinence and dysphagia, patients endorsed also dyspepsia, early satiation, postprandial fullness, bloating, belching, globus sensation and abdominal pain. Additionally, irritable bowel syndrome was noted. Degree of disability, urinary dysfunction and female gender have been found to be independent predictors of anorectal dysfunction [112,113]. Age and primary progressive MS were also shown to be associated with bowel symptoms [112,114].

### 5.3. Background

Etiology of GI symptoms is multifactorial; immobilization and polipharmacy may have a causative role in the development of GI symptoms in MS patients [115]. Coincidental pelvic nerve lesion during childbirth could also underlie fecal incontinence in women with MS [115]. Spinal cord lesions appear to be most important in the pathogenesis of GI symptoms in MS. The pathways of neural control of defecation are not fully defined, however, cortical and pontile centers may play a pivotal role in the regulation of sacral segments [116,117]. Conduction times of central motor pathways to sphincteric sacral neurons and pelvic floor striated muscle have been shown to be prolonged in MS [118,119]. Impaired anorectal sensation may also underlie the symptoms: somatosensory evoked potentials were delayed in MS compared to controls [120]. Loss of central modulation on spinal cord segments may lead to sympathovagal imbalance which can lead to constipation, characterized by lengthened colon transit time, lack of increment in colonic motility postprandially, early sphincter excitation when rectal filling, and increased threshold for anorectal reflex [116,121,122]. Constipation has been attributed to rectal outlet obstruction, absent or incomplete puborectal, anal canal and sphincter musculature relaxation and reduced voluntary anal squeeze pressure [118,123,124]. In fecal incontinence, studies have described reduced sensation of rectal filling, reduced rectal compliance, low anal sphincter pressures, hyperreactivity of the rectal wall [121,125]. Many patients with MS have reduced maximal voluntary anal sphincter pressures [108]; hence they cannot delay defecation by contracting the sphincter, as would be possible in health. The coexistence of fecal incontinence and constipation can be explained by similar mechanisms described by DSD. Incoordinated action of the external/internal anal sphincter during expulsion, poor pelvic musculature performance may cause incomplete emptying of the rectum, which precipitates fecal incontinence when anal sphincter weakness and anorectal hyposensitivity is present [118,121,124,126]. There is a lack of data on the background of upper GI symptoms. Gastric emptying rate has been found to be abnormal in MS patients; however, the study did not correlate upper GI symptoms and gastric motility [127]. Further research appears needed to clarify etiology of bowel dysfunction, correlating imaging, physiological studies, EDSS scores and bowel symptoms.

### 5.4. Assessment

There is no established diagnostic algorithm for bowel symptoms in MS. There is no bowel symptoms questionnaire validated specially for MS. The physician, after the detailed clinical interview may choose the Neurogenic Bowel Dysfunction Score, which has been designed for spinal cord injury patients to evaluate large bowel dysfunction [128]. Other possibilities include the Adult Functional GI disorders Rome III Questionnaire [129], probing the presence, severity and duration of different GI symptoms, the MD Anderson Dysphagia Inventory to detect emotional, functional, physical symptoms related to deglutation [130] and the Fecal Incontinence Severity Index [131]. Several studies use definitions of national or international guidelines to determine existence and severity of GI symptoms, such as the consensus of the American Gastrointestinal Association. Collaborative referral with GI specialist may be warranted. Beyond questionnaires, anorectal physiological measurements can be performed, such as colonometry, anal manometry to measure contraction pressures, balloon distension or mucosal electrosensitivity test to quantify rectal sensation, defaecography/proctography to analyze defaecation dynamics, gastric scintigraphy, colon transit studies to determine emptying rates with the help of swallowed markers or pudendal nerve terminal motor latency test to gain more comprehensive overlook on neuromuscular cooperation [123,127].

### 5.5. Treatment

Nonpharmacological as well as pharmacological treatments are available for constipation (increasing activity, drinking more fluid, mechanical evacuation, bulking agents, osmotic and stimulant laxatives, rectal stimulants, biofeedback, prokinetic agents, and rarely colostomy, Malone procedure) [107,132]. Patients with fecal incontinence are provided with fewer therapeutical options (antimotility drugs, biofeedback, sacral nerve stimulation, surgical techniques: sphincter repair, dynamic graciloplasty, artificial bowel sphincter) [107,132]. Only few trials tested the efficacy, acceptability of these aforementioned interventions in patients with MS. Gut focused behavioral treatment appears to influence GI symptoms in MS favorably [133]. A small study (*n* = 30 patients) showed significant descent of constipation scores when MS patients received abdominal massage [134]. Transanal irrigation has been recently found successful in a prospective observational study of MS patients with bowel symptoms [135]. In spinal cord injury and Parkinson’s disease, fiber (psyllium) [136], oral laxative (macrogol electrolyte solution) [137], parasympathetic stimulant neostigmine-glycopyrrolate [138], rectal stimulant polyethylene glycol [139] have been found to be useful. Based on the similarities between central neurological diseases, one could hypothesize that these therapeutic strategies may bring substantial results in the management of MS patients with bowel symptoms.

### 5.6. Perspective

A new proposal suspects altered gut microbiome as a source of chronic inflammation that could lead to the development of MS [140]. Pathogens such as *Helicobacter pylori* may access the central nervous system via fast axonal transport by afferent neurons connecting the GI tract to the brain [141]. Studies investigating the association between *H. pylori* infection and MS brought contradictory results, however, in a pilot study successful *H. pylori* eradication resulted in lower second episode occurrence and a significant improvement of the EDSS score in clinically isolated syndrome patients after the two year follow-up [142,143,144]. Taken together, there might be a possible bidirectional relation between GI problems and MS development, which emphasizes the importance of further research on this field.

## 6. Sexual Dysfunction

### 6.1. Epidemiology

Sexual dysfunction (SD) is a highly prevalent but often underdiagnosed symptom of multiple sclerosis. It affects the quality of life negatively and may reduce fertility [7,145,146]. For men with MS, the reported rates of SD range from 50% to 84% [147,148], while 34% to 85% of female patients suffer from sexual dysfunction [146,147]. The most common complaints for men are erectile and ejaculatory dysfunction, occurring in 20%–70% and 13%–53% of male patients with MS, respectively [147,148,149]. For female patients, the most prevalent symptom is decreased sexual desire (31%–58%) [147,150,151]. SD can emerge at various stages of the disease: at an early phase of the disease, with minimally affected patients [152,153], but also between 2 and 6 years after diagnosis [154]. The etiology of SD in MS patients is complex including both organic and nonorganic pathomechanisms. Consequently a multidisciplinary approach is required to diagnosis [155].

### 6.2. Complaints and Associations

Sexual problems in MS can stem from primary, secondary, or tertiary sources [156].

Primary SD refers to the direct result of MS due to demyelinating lesions in the nervous system. Irrespective of gender, symptoms may include numbness or sensory paresthesia in the genitals, reduced libido and orgasmic dysfunction [146,157]. For men, erectile dysfunction (ED) and ejaculatory dysfunction are the most concerning symptoms [147]. Men with ED may still experience penile tumescence during the night and erections when waking up [158,159]. Ejaculatory dysfunction may take different forms, including premature ejaculation (up to 60%), delayed ejaculation (up to 50%), retrograde ejaculation, anejaculation (up to 33%) [160]. Decreased vaginal lubrication, impaired clitorial erection, dyspareunia are gender specific problems for women [149,161,162].

Secondary SD means that non-sexual MS-related physical alterations eventually affect sexual functions adversely. Very frequently occurring MS-associated problems, such as fatigue and neurogenic bladder symptoms were found to amplify SD in both sexes [146,150,163], but women appear to be more affected [149,164]. Spasticity, pyramidal signs in the lower limbs, pain, paresthesias and reduced mobility have been reported to correlate with SD [165,166,167]. Controversial associations were found between gastrointestinal symptoms and SD [147,148].

Tertiary consequences correspond to psychosocial, emotional and cultural problems that interfere with sexual experience. These problems include social role changes that originate from MS, changes in self-perception, feeling of being less sexually attractive, and worries about sexually satisfying the partner and communication difficulties [148,157,168]. Increased positive support from the partner has been found to improve sexual satisfaction, while negative support decreases satisfaction [168]. Depression, which occurs in more than 50% of elderly MS patients [169], and anxiety are significantly associated with SD irrespective of gender [147,170,171,172]. Positive correlations were described between higher economic status, education level, cognitive performance and sexual function in MS patients [154,170,171].

Level of disability as measured by EDSS as well as age, and disease duration are correlated with SD in female MS patients [148,149,161,173,174]. Advancing age and age at onset of MS appear to be associated with SD in men, while level of disability seems to be less influential [146,147,170]. Side effects of medication currently used in the clinical practice may have the potential to impair sexual functioning. Antidepressants, such as selective serotonin reuptake inhibitors, may cause delayed ejaculation, absent or delayed orgasm, reduced desire and difficulties at arousal [175] and they also lead to a significant deterioration to female sexual function [176]. Others have found that anticholinergic medications enhance sexual problems for women [177].

### 6.3. Neurohumoral Mechanisms

Demyelinating lesions in the parasympathetic sacral spinal cord have been linked to erectile dysfunction in men [166,178]. Central nervous system demyelinization, e.g., pontile plaques may also cause erectile disturbances [170]. Erectile dysfunction has also correlated linearly with reduced serum testosterone levels and impaired hypothalamic–pituitary–thyroid axis function [179]. Damage of the sympathetic thoracolumbar segments in MS may cause abnormal ejaculation [170].

In women, brain stem, pyramidal lesions and pontile parenchymal atrophy showed associations with SD [170,180,181]. Hormonal alterations, such as high levels of prolactin, luteinizing hormone (LH) and follicle-stimulating hormone (FSH) were reported in women with MS, which were thought to exist due to peripheral resistance to gonadotropins, abnormal central regulations and immunosuppressive therapy [182,183]. In a recent cross-sectional study, only 10% of MS women had low beta estradiol, 7% had low progesteron plasma concentrations. Testosterone levels were within normal range for female MS patients but the subgroup of patients with sexual dysfunction had significantly lower serum testosterone concentrations [184].

### 6.4. Assessment

Patients are usually embarrassed to talk about their sexual problems; therefore, physicians should address the topic and provide patients with information on the available different treatment options. To initiate assessment, comprehensive clinical interview should identify the SD, and then standardized questionnaires are to be used to explore SD in precise details [168]. Multiple Sclerosis Intimacy and Sexuality Questionnaire (MSISQ-19) is a valid, reliable, self-report questionnaire for men and women, which assesses all three domains of SD [156]. The shorter form of MSISQ-19 became a 15-item questionnaire, which can be handled easier by the cognitively defectualized patients [185]. Sexual assessment has also used functional paradigms or personal satisfaction experience using the Sexual Satisfaction Survey in particular [186]. Gender specific questionnaires include Female Sexual Function Index and International Index of Erectile Function [187].

Other tools include Quantitative Sensory Testing, which assesses genital neurological deficits related to the detection of temperature and vibration [188]. Diminished pudendal somatosensory evoked potentials were also found to be associated with SD [189].

### 6.5. Treatment

Sexual dysfunction in MS requires interdisciplinary urological and neurological expertise and should therefore preferably be treated by specialists.

#### 6.5.1. Primary Sexual Dysfunction (SD)

Oral phosphodiesterase type-5 inhibitors (PDE5i) are considered first-line treatment in patients with ED and central neurological disorders [190]. Double-blind, placebo-controlled randomized study of sildenafil demonstrated significant amelioration of erectile, orgasmic, overall sexual function in a large number of MS patients, ranging from 73% to 95% [6]. Results on tadalafil are comparable to sildenafil and it may be favored in selected patients due to its longer duration of effect and lack of interaction with fatty meals [178,191]. PDE5i are especially effective for patients with upper motor lesions, having erections on the morning waking [190]. Second line treatment for ED involves intracavernous injections of prostaglandin E1, which was found to be effective in men with MS [159,192]. Patients with lower motor neuron lesions lacking reflexogenic erections may benefit from this treatment. Dopamin receptor agonists (e.g., sublingual apomorphine), vacuum constriction devices or penile prosthesis can be further possibilities for some patients [132,193]. Oral midodrine therapy was found to restore ejaculation in some MS patients [194]. Penile vibratory stimulation appears to be an effective treatment for ejaculatory problems in MS, as well [195].

There is a lack of data on the management of SD in women with MS [132,196,197]. PDE5i, such as sildenafil was reported to improve lubrication, but not the ability to reach orgasm [198]. However, when PDE5 inhibitors were combined with antidepressants there was a marked improvement in orgasmic function, as well [199]. Low-dose local estrogen has been found to improve clitoral sensitivity and reduce dyspareunia [132]. Estrogen applied together with methyltestosterone has been suggested to intensify sexual desire and vaginal lubrication [132]. A recent interventional study indicated that pelvic floor muscle training alone or in combination with intravaginal neuromuscular electrostimulation or transcutaneous tibial nerve stimulation improves SD [200]. The efficacy of α-1/2 receptor antagonists on SD in MS patients still remains to be elucidated.

#### 6.5.2. Secondary SD

Education and advising by the healthcare providers may bring significant relief for MS patients. Suggestions such as planning sexual activity in the early morning hours, convenient sexual position, performing bladder catheter before sexual activity, not putting pressure on the bladder during the activity can help to limit the effect of fatigue, spasticity and bladder dysfunction on sexual experience [154,170]. OnabotulinumA injection therapy was also found to affect positively SD, probably by improving urinary dysfunction and depression [201]. MS treatment should counteract sexual functioning minimally. Discovering and treating mood disorders is crucial. However, because of the side effects of antidepressant drugs, physicians may consider lowering the dose, or prescribing other antidepressants with lower risks of sexual dysfunctions (e.g., bupropion, reboxetine) or adding a serotoninergic antagonist (e.g., mirtazapine, mianserin) to the drug therapy after careful assessment of the benefits and risks of each drug [199].

#### 6.5.3. Tertiary SD

Therapeutic interventions include general/couple support counseling and psychotherapy [173]. The aim of therapy should be pleasure and satisfaction, rather than achieving a perfect genital response [202]. Sexual counseling should include learning about extra-genital areas that can be stimulated to provide erotic sensations. Psychotherapy has been shown to improve communication and sexual satisfaction markedly [168]. Interventions, especially with female MS patients should emphasize the fact that, despite the disability, patients remain sexual beings who can love, bond, accept and donate joyful experiences [203].

#### 6.5.4. Fertility Issues

Limited studies have investigated semen quality in MS patients. In MS patients, semen quality has been found to be impaired compared to healthy volunteers [179]. It has been reported that fertility, defined as the number of children that are born to women with MS, is reduced [145,204]. Other clinical observations in MS patients indicated that ovarian reserve is diminished and antimüllerian hormone level is lower, hence fertility is reduced [205,206]. Side effect of medication, especially immunomodulatory therapies may further impair fertility. Advanced methods of assisted reproduction may be considered in MS patients with SD refractory to the aforementioned treatment strategies [207].

## 7. Sudomotor Dysfunction

### 7.1. Epidemiology

Sweating impairment is a well-known complication of MS [208,209,210]. Several diagnostic and epidemiological studies examined sudomotor dysfunction in MS. The observed prevalence range is wide which may in parts be caused by heterogeneity of diagnostic repertoires and definitions of sudomotor abnormalities applied in these studies. Pathologic sweating tests ranged between 26% and 94% [211,212,213,214].

### 7.2. Clinical Manifestations

Decreased sweating response or regional anhydrosis to thermal provocation are the most common clinical manifestations of sudomotor dysfunction [209]. Besides, total anhydrosis could appear in advanced MS [209]. Impairment of sudomotor function in MS patients is correlated to the severity of clinical disability [208,211,213] and may cause disturbances in thermoregulation [215]. Appropriate functioning of the theromoregulatory apparatus is crucial in patients with MS because slight changes in core temperature could aggravate neurologic symptoms [208,216] due to heat induced conduction abnormalities of demyelinated axons [217,218,219]. Physical exercise and increased ambient temperature are the most important heat challenges for MS patients [210].

### 7.3. Pathology

The damage of the central sudomotor pathways caused by demyelination has been suggested the most important pathological pathway of sudomotor dysfunction in MS [209]. The major components of the central descending sudomotor system are the preoptic region of the hypothalamus, the tegmentum of the pons, the lateral reticular substance of the medulla and neurons in the intermediolateral column connected with sweat glands through sympathetic ganglia and peripheral nerves [215,220]. Additionally, total lesion volume of the brain and lesions found in thoracic spinal cord registered by MRI have been linked to impaired somato-sympathetic sweat reflexes [211]. Impaired sweat gland function can also be caused by deteriorated sympathetic input leading to sweat gland atrophy, reduced sensitivity to cholinergic stimulation and muscarinic receptor down-regulation [221].

### 7.4. Assessment

Clinical testing of sudomotor function can be performed by means of thermoregulatory sweat testing (TST), quantitative sudomotor axon reflex testing (QSART), sympathetic skin response (SSR), and quantitative direct and indirect test of sudomotor function (QDIRT) [84,222]. Combined use of these assessment techniques can localize and quantify pre- and post-ganglionic sudomotor lesions and thus improve accuracy of diagnosis and disease monitoring in autonomic disorders. In MS, sudomotor function assessment has not yet been widely established in clinical practice. So far, TST and SSR have been widely used for testing sudomotor autonomic regulation in MS [208,209,211]. TST using indicator powder mixture provides semiquantitative estimation of sweating function [215]. Sudomotor function can be quantified by TST complemented with evaporimetry [208,223]. TST is used for the evaluation of pre- and post-ganglionic axon function. Measurement of sympathetic skin responses gives information about sweating caused by somato-sympathetic reflexes. The technique shows high sensitivity but is limited by interindividual variability [211,213,215,223]. Examination of the number, size and volume of sweat droplets after direct chemical stimulation by pilocarpine iontophoresis can characterize eccrine sweat gland function [215,221]. Post-ganglionic sudomotor function can be specifically assessed by evoking an axon reflex in cutaneous sympathetic small nerve fibers. The sudomotor axon reflex can be induced by cholinergic agonists binding to nicotinic receptors on the terminals of sudomotor nerves. The evoked action potentials are conducted antidromically to an axon branch point and then orthodromically to a neighboring population of eccrine sweat glands to induce indirect sweating in a skin area outside the stimulation area. In contrast to indirect axon reflex sweating, the direct response is induced by stimulation of muscarinic receptors located on sweat glands. Direct and axon-reflex-mediated sweat responses can be evoked by iontophoretic application of acetylcholine [224]. The QSART assesses post-ganglionic sudomotor axon reflex mediated sweating following iontophoresis of acetylcholine with temporal resolution [215,225]. QSART is a sensitive, reproducible and accurate quantitative method [215]. QDIRT measures both direct and indirect sudomotor responses with spatial and temporal resolution. This experimental technique has not yet been established in clinical practice [226]. In addition to QSART, QDIRT might be an excellent new option for sudomotor function testing in MS patients but prospective data is still lacking to support this hypothesis.

### 7.5. Treatment

The most important task for patients with sudomotor dysfunction is to minimize heat exposure. Outdoor work and physical exercise should be performed early in the morning or after sunset [210]. Precooling was found to have favorable effects on minimizing newly onset symptoms related to physical activity [227]. Cooling of the head and the neck can provide an appropriate reduction of oral and body temperature for symptomatic relief of heat-induced symptoms [228]. In addition, cooling garments serve as a suitable tool to alleviate the deleterious effects of heat challenges [229,230]. Moreover, potassium channel blockers could have favorable effects on axonal conduction during heat provocation [231].

## 8. Pupillomotor Dysfunction

### 8.1. Epidemiology

Pupillary disturbances are prevalent complications of MS. Prevalence of abnormal pupillary responses ranges between 26% and 60% in patients without a recent history of acute optic neuritis (ON) [232,233]. The time difference between the ON and the measurements was at least 6 months in these studies. ON is a common clinical feature during the course of MS often connected with relative afferent pupillary defect [234,235].

### 8.2. Pathophysiological Characteristics

No associations were found between pupillary abnormalities and disability of patients quantified by the EDSS or duration of the disease [236]. Pupillary abnormalities in MS might be due to non-specific impairment of central pathways subserving pupillary function. [237]. Pupillary disturbances and visual abnormalities do not change parallelly during the course of the disease [232,233,236]. Patients with completely recovered visual acuity can have pronounced pupillary dysfunction during the convalescence after acute optic neuritis [238]. The most severe disturbances were found in patients with primary progressive MS in a study published by De Seze *et al.* [232]. Pupil color response (PCR) abnormalities are usually more pronounced than pupil light response (PLR) deficits in the active phase of demyelination, while PLR abnormalities are more common than PCR deficits during the recovery phase [238].

### 8.3. Background

Autonomic pupillary dysfunction has been shown to be the consequence of sympathovagal imbalance. Decreased parasympathetic tone associated with increased sympathetic tone results in pathological pupillary responses in MS patients [232,236,237]. Impairment of either afferent or efferent pupillary pathways was demonstrated by several studies [232,233,239]. Axonal loss seems to be a more important determinant of pupillary abnormalities than demyelination according to MRI studies [232,237]. Pupillary light reflex metrics are associated with retinal ganglion cell axonal degeneration assessed by average retinal nerve fiber layer thickness [240].

### 8.4. Assessment

Pupillary reactions can be examined with computerized pupillographic devices. The most frequently measured parameters in MS patients assessing parasympathetic function are the pupillary light reflex latency (PLRL), reflex amplitude, contraction velocity, while the parameters representing sympathetic function are the redilatation velocity, time of 75% redilatation and redilatation at 5 s [233,236,237,241]. The PCR is also measureable by similar pupillographic equipment with the application of special stimulation with colored targets [238]. It is possible to distinguish between the damage of afferent and efferent pathways based on the measurement of direct and indirect PLRL [242]. The swinging flashlight test used with neutral density light filters is a simple method for the semiquantitative determination of relative afferent pupillary defect at the bedside [243]. A more objective, computerized form of this test with the determination of second flash pupillary metric response asymmetry ratios provides an accurate tool for the measurement and follow-up of relative afferent pupillary defect in patients with MS and ON [235]. Measurement of pupillary functions can provide clinical data on sympathovagal imbalance, demyelination processes, retinal ganglion cell degeneration during the follow-up of MS patients. Therefore, pupillary function assessment may be a useful technique to complement diagnostic tools of cardiovascular, urogenital and gastrointesinal function in clinical practice. However, confirmatory studies in large populations are lacking to support the usefulness of pupillary function assessment in MS.

## 9. Limitations

This is a review of the current literature which neither intended to be exhaustive nor to compare or evaluate efficacy of treatment regimens of autonomic disturbances in MS. Both the diagnostic tools and treatment regimens included in our review are, in parts, based on heterogeneous evidence including both randomized and nonrandomized studies. However, our review might provide a helpful overview of the clinically relevant field of autonomic dysregulation in MS and may form the basis for a synthesized data analysis comparing diagnostic and therapeutic regimens.

## 10. Summary and Perspective

Dysregulation of the autonomic nervous system may present with various clinical symptoms in MS patients that reduce quality of life. Prevalent and clinically relevant autonomic disturbances include neurogenic lower urinary tract dysfunction, symptoms of cardiovascular and gastrointestinal dysregulation as well as sudomotor and pupillomotor dysfunction. These disturbances constitute a clinical challenge to the physician due to variability of clinical presentation and inconsistent data on diagnosis and treatment. Furthermore, the mechanisms whereby autonomic disturbances are mediated in MS are not fully elucidated, impeding the development of causative treatment. In clinical practice, early diagnosis and initiation of individualized interdisciplinary and multimodal strategies appear to be beneficial in the management of autonomic dysfunction in MS. Further research is warranted to improve our mechanistic understanding of autonomic dysregulation in MS, improve precision of diagnosis and assessment of response to treatment, and improve strategies of both causative and symptomatic treatment.
